# Migraine

**Published:** 1895-01

**Authors:** Hugh Hagan

**Affiliations:** Atlanta, Ga.


### MIGRAINE.*

BY HUGH HAGAN, M.D.,

ATLANTA, GA.

The honor of presenting to so honorable and distinguished a body my ideas on the subject of migraine is greatly appreciated, and I sincerely hope you may be repaid for your time and kind attention. Should I be able to make one useful suggestion, or one that you may deem wise to apply, I will be more than repaid.

The subject of this humble effort is known by the following names: Migraine, megrim, hemicrania, nervous headache, sick headache, and bilious headache. This disease is nosologically classified as a neurosis. My interpretation of a neurosis is a derangement of the nervous system, the pathology of which is as yet unknown.

As far as modern pathology goes the neuroses are irritable or reflex manifestations of a disorganized cerebro-spinal integrity.

Having then a subject of unknown pathology with which to deal, we are compelled to estimate the value and importance of both objective and subjective symptoms according to the lights at hand. This being the case, our first step should be to discover the predisposing and exciting causes of this most trying and frequent disease.

Believing in the infinity of the range of possible malevolent influences on the human organization and in the immutability of the laws of atavism, I deem it absolutely necessary that as thorough and complete a history of the family record, of the hygienic, the dietetic, the moral, and the vocational life of the patient be elicited as is possible.

No organ should be left unexplored, for it might be that some more careful brother will discover that which was overlooked or considered as a trifling irregularity to be the seat of the reflex irritation.

---

*Read by title before the Tri State Medical Society, Atlanta, October, 1894.

Of the many accepted predisposing and exciting causes of this trouble, we will first occupy your attention with the more important or predisposing ones.

The most frequent cause of migraine is heredity. Most authorities estimate that over fifty per cent. of all cases can be traced to some neurotic taint or organic cerebral spinal or cerebro-spinal defect in the immediate family of the sufferer from migraine. It need not be a direct inheritance of migraine but an hysteria, a mania, or any form of insanity, an epilepsy, or in fact any organic or functional derangement of the cerebro-spinal nervous system of grandfather or grandmother, father or mother, or aunts or uncles. Generally the weakness is transmitted through the maternal side, but also through the paternal and collateral branches.

Next in importance as to frequency and potency are errors of refraction, or of muscular insufficiency. The most common errors of refraction are hypermetropia, and of muscular insufficiencies, exophoria and esophoria.

In regard to the frequency and importance of ocular defects in persons suffering from migraine, Dr. G. T. Stephens, of Brooklyn, and Dr. Ranney, of New York, have ably and exhaustively treated, and I would advise any one of you gentlemen present, who has not done so, to read these gentlemen's ideas on the subject.

I myself have seen great benefit from the correction of errors of refraction and of muscular weakness in sufferers from migraine, and I have in over ninety per cent. of my patients found some error in refraction or in muscular tone, to a greater or less degree, and always observed benefit from the correction of the same.

Next in frequency to ocular defects as a causative factor in the production of migraine are gastric troubles, especially chronic gastric catarrh and the disturbances arising from indiscretions as to the ingestion of indigestible articles of food and alcoholic beverages. As most of the indigestible articles of food and alcoholic beverages are baneful at best, they are more baneful in consequence of the existing catarrh.

Fourth in importance as an etiological factor is sex. More females than males suffer with this trouble. Whether this be due to the many and frequent derangements in the generative organs in the female, or to her acknowledged less stable organization, I am not prepared to state, but the fact remains.

Next to sex comes age, more cases beginning in early manhood or womanhood than in childhood or middle or advanced life. I doubt not but that the period of evolution at puberty is a time most liable for a weakness to show itself. Generally at the climacteric period in the female, or the homologous period in the male, the frequency and severity of the attacks are lessened and disappear.

Following age as a frequent cause are the gouty and rheumatic diatheses. I have been able in a great number of cases to ascribe the attacks to these conditions.

From this point on we may mention with equal emphasis as predisposing causes, diseases of the middle ear, of the nose, congestion of the liver, constipation, fright or any severe emotion, tabes dorsalis, violent concussion, anemia, plethora, overwork (especially mental), malaria, lithemia, and sympathetic derangements, whether angeiospastic or angeioparalytic. The possible exciting causes are so numerous that I will only mention a few of the most frequent: Exposure to the elements, crowded assemblages with the consequent bad ventilation, railway travel, sudden barometric and thermometric changes, long hours of close application, and fatty and highly seasoned food. Often no cause can be ascribed other than a seeming periodicity.

The treatment of migraine consists of the management of the individual attacks or in combating the constitutional weakness.

Dr. Gray, in his work on the "Diseases of the Nervous System," states that it may be accepted as an axiom, our inability to abort an attack; but as I have had the pleasure of a seeming abortion in several of my patients, I am inclined to think this statement of Dr. Gray's too sweeping, and am persuaded to advise all to make an effort to abort an attack, as I deem it greatly to the patient's advantage to break up the habit of pain, and especially to lend every effort at our command to destroy the periodicity, so often manifested in the appearance of these headaches. Believing the above to be true, I think it wise to prescribe whatever drug may have been found to benefit any one particular case, with the desire of aborting the attack, for granting the possibility of failure in this special aim, we have the consolation of knowing that if not abortive, the drug may prove palliative.

After considerable experience in the management of these especial forms of headache, I have found that each attack differs in some slight detail from the preceding, and what may have proven beneficial for two or three such attacks will be found wanting in good effects afterward. So as there exist differences in attacks in the same individual, there exist greater in different individuals. So we are constrained to vary our prescriptions accordingly. As a general thing, as an abortive, in pending attacks, we will find that a brisk purgative, such as a five or ten-grain dose of calomel and rhubarb, or calomel and jalap, with ten or twelve grains soda bicarb. Accompanying this purge, antipyrin in five or ten-grain doses every half-hour or hour for three or four hours will, in many cases, prove beneficial. Other drugs, such as antifebrin in two and a half or five-grain doses, phenacetine in five or ten-grain doses with an alkali, administered every half-hour or hour, will render signal service. Of course in the administration of these drugs their depressing effects must be remembered, and also to give them preferably in solution or powder, not in capsules or compressed tablets, as much time is often lost by the insolubility of the gelatine, or the time necessary to disintegrate and dissolve the tablet. These little points are well worth remembering. I have been in the habit of giving the following prescription, most always accompanied by a purgative, to wit:

| | |
|---|---|
| Phenacetine | gr. v. |
| Caffeinæ citrate | gr. ii. |
| Sodii bicarb | gr. v. |

Repeated every half hour until four or six will have been taken, and generally with gratifying effects.

Next in importance to the above mentioned suggestions as palliatives, come the bromides. A full dose of either of the bromides will often relieve an attack to a wonderful degree, and especially if the attack be of the angeioparalytic type, twenty or thirty grains of the bromide of potassium or sodium, with a drachm of Squibb's fluid extract of ergot, will do wonders. If the attack be characterized by a pallid countenance, with cold sweat and cold extremities, constituting the angeiospastic type, $\frac{1}{100}$-grain doses nitro-glycerine, either by mouth or hypodermically, are to be recommended. The nitrite of amyl, three to five drops, inhaled every ten or fifteen minutes for two or three times, will be found potent for good. This last mentioned drug causes at times a very uncomfortable feeling of fullness in the head and a sensation of oppression around the heart, but I have never seen any ill effects follow its administration. At times the severity of the pain and the great prostration will call for the administration of morphine. I never hesitate to administer this drug if I deem it advisable, though I consider the danger of forming the morphine habit on account of the chronicity of the disease, and the pardonable desire of the sufferer to resort to any means for relief. I never allow myself to be influenced by the popular or vulgar verdict that the doctor is often responsible for the formation of this habit. My courage in administering this drug is fostered by the conviction that in conditions characterized by great pain, a superlative tolerance of opium is often manifested, and that for fear of being adversely criticised my patient might be deprived of relief not otherwise to be obtained, so that thus far in my career, all things being equal, I give my patient the benefit of whatever drug I may decide will meet most of the then existing exigencies. Unlike opium, the next drug that has the power to relieve pain is accompanied with such ever-present danger to life, that I always endeavor to resist administering it. I refer to chloroform. I think in the vast majority of cases which are severe morphine will do all that it can do, and we will avoid the depressing cardiac and respiratory depressing effects.

It is a common thing to meet with cases suffering from migraine which, from the constant inhalation of chloroform, have so injured themselves that I considered the means of relief had been a thousand-fold more injurious than the ill effects of oft-repeated attacks of the headaches. On this account I never use it, and as I do in administering most dangerous drugs, make emphatic my objections against the patient taking them independent of medical advice. Our list of palliative drugs does not end here. The fluid extract of guarana is highly recommended, and for the few times that I have prescribed it, I am inclined to believe that in fifteen or twenty-drop doses much good may be noted.

Large doses of an alkaline mixture, such as half an ounce of the spirits of the acetate of ammonia, or a draught of hot water with tea-spoonful of carbonate of soda, Vichy water, or any of the natural alkaline mineral waters, are at times efficacious. The liquor ammonii acetate with sodium salicylate is good. Hot baths or hot applications to the head, in conjunction with the above, are also grateful. The menthol pencil or the crystalline liniment of Wyeth is a good subefacient and revulsive. This latter consists, I think, of menthol and oil mustard. I also advise, in conjunction with the above mentioned means of relief, rest, quiet, darkened room, and electricity. Electricity in its various forms has been used extensively in Germany as a palliative treatment in migraine, and of later years has come much in vogue on the continent of Europe, England, and somewhat in this country. The application of electricity differs as to the especial mode in which it is to be administered, according to the nature of the current used. So we will consider separately the different currents.

Erb recommends the galvanic, or constant current, in preference to the faradic or induced, or the frictional.

In applying the galvanic current, the operator should be certain that his battery is steady, that his milliampère meter is in good order, that his rheophores and electrodes are in perfect trim, and he should be master of the details necessary to administer intelligently this subtle and potent fluid, for if not well done, 'twere better left undone. Being certain that all is in readiness, the electrodes should be moistened with an alkaline solution, thereby lessening the resistance offered by the patient to the current, and one placed on the forehead and the other on the nape of the neck, and the current slowly turned on. The current should be from two to five milliampères, and always short of producing the sensation of dizziness. After the passage of the current anterio-posteriorly three or four minutes, gently and uninterruptedly turn off the current and place the electrodes over the mastoids. On no account ever interrupt the galvanic current when applied to the brain, unless it be for diagnostic purposes in diseases of the middle ear.

The total length of time in cerebral galvanization should not exceed ten minutes, as a general thing.

The application of the faradic current is also frequent in this disease, and is to be recommended.

There are also several methods of applying the secondary or induced current, either the sponge electrodes, the faradic hand, or the wire-brush electrode. Each method will at times give relief. The direct application of the moistened electrodes is the same as when applying the galvanic current, and the current strength is to be regulated by the patient's sensation, it never to be sufficient to produce decided pain. As yet we have no absolute meter for gauging the strength of the faradic current.

The faradic hand consists of simply interposing the operator's body between the electrode. The operator holds one electrode in one of his hands, the patient the other, or keeps the electrode in place on some part of his body, preferably the nape of his neck, then the operator's free hand is moistened and applied to the patient's forehead, or over cervical sympathetics.

The wire-brush electrode is quite severe, and I never use it. As to the benefits of the Franklin machine, I have very slight knowledge, save from reading, as I never use it. Many authorities speak of its benefit with great praise.

Now the important task confronts us of curing the constitutional weakness. It is a mooted question if our best efforts are palliative instead of curative. It is a deplorable fact that though the frequency and severity of the individual attacks can be lessened, an absolute cure—and by absolute cure, I mean that after we shall have succeeded in warding off the spells for such a length of time as to justify us in discontinuing our attentions, that they will never more return—is not to be promised. I fail to find recorded, and I have certainly not achieved an absolute cure.

The treatment of migraine, with a view of improving the condition, consists of medicamentation, dietetics, hygiene. First of all, any error of refraction, be it never so slight, should be corrected, for modern literature abounds with favorable reports following the use of spherical and prismatic lenses for hypermetropia, and exophoria, esophoria, or hyperphoria, respectively. Abnormal conditions of nose, ear, and throat should receive proper attention. Gastric catarrh should be combated by rigid nitrogenous diet, cadage, the alkalies, and bismuth or cerium, nux or other bitters; anemia by cold baths, massage, general faradization, full diet, large doses of iron, either the ammono citrate in ten-grain doses, the citrate of iron and quinine, dose, grains ten, the tincture of the chloride, grains ten to fifteen, the dialysed and the albuminate in one-grain doses, well diluted in water, the peptonate in three to five-grain doses, or Bland's pills. Small doses of the corrosive chloride of mercury added will prove beneficial in sea travel. The mineral acids, especially in cases caused by lithemia, will most always give satisfactory results, though at times we will find the alkalies do better, and even best of all when given an hour or two after meals.

The drug par excellence in the treatment of migraine is cannabis indica, it reaching almost the dignity of a specific. Without exception the modern writers agree in the great and favorable results of this drug, either in the form of a fluid or solid extract,—generally the solid extract in from one-eighth to one-third grains, three times a day for months, with the proper diet and hygiene; this drug is the sheet anchor and hope of the sufferers from migraine, though at times alarming effects are produced by very small doses. I have a patient who possesses an idiosyncrasy so susceptible that the effect of one-sixth of a grain of the extract produced such alarming effect I had to desist in my efforts in trying to administer it. A small dose of one of the bromides at night will add to the efficacy of the cannabis. Mild laxatives or an occasional calomel purge will do much to relieve constipation and engorged livers and turgid hemorrhoidial veins. Out-door exercise, long hours of rest, sea voyages, and sojourns in different climates have all, at times, a happy influence.

This, gentlemen, embraces my ideas on the subject of the etiology and treatment of migraine in as few words as I could express them.

209 *Peachtree St.*

